# Balancing autonomy and expediency within legal parameters: providing primary care to unaccompanied minors

**DOI:** 10.1186/s13584-018-0241-0

**Published:** 2018-07-30

**Authors:** Kelly Orringer

**Affiliations:** Michigan Medicine, 300 North Ingalls #6E12, Ann Arbor, MI 48109 USA

**Keywords:** Unaccompanied minors, Adolescent, Primary care, Communication, Health law

## Abstract

The issue of how primary care clinicians manage unaccompanied minors is not well studied. This month’s article “Treatment of unaccompanied minors in primary care clinics- Caregivers practice and knowledge” begins to fill that gap. The study results reveal that Israeli primary care nurses and doctors often treat unaccompanied minors. Legal parameters offer significant latitude for urgent or simple and ordinary care. Communication to parents afterward is inconsistent. Clinicians also appear to be operating without full understanding of the law in this regard.

This contrasts somewhat with the American situation wherein state level laws more clearly proscribe what types of treatment may be offered to adolescents without the prior consent of a parent and also what may remain confidential. Also, in the US, the variability of what is permitted varies widely across the 50 states and territories.

The tensions between offering appropriate and timely care, maintaining the trust of patient and family, and doing what is expedient are all important considerations for primary care clinicians who treat unaccompanied minors. This exploratory study identifies current Israeli practice and should serve as an invitation to other national primary care groups to examine their own current state and work towards best practices.

The Israel Journal of Health Policy Research (IJHPR) has recently published an article entitled “Treatment of unaccompanied minors in primary care clinics – Caregivers practice and knowledge [1]” which details how clinicians handle this issue in practice in the context of Israeli law and societal norms. This is one of the few studies in the literature to directly query clinicians to determine what occurs in primary care practice when a minor patient presents for care without a parent or legal guardian and how this conforms to applicable national laws. The authors’ findings are highly relevant to pediatricians and others who deliver medical care to children.

Peled-Raz, Perl, and Green’s cross-sectional study [1] surveyed 158 primary care doctors (*n* = 55) and nurses (*n* = 103) in the Haifa and Galilee districts of the Clalit Health Services. The responding clinicians were caring for rural and urban youth of Jewish, Arab, and other backgrounds across socio-economic strata. The aim of the study was to determine both actual practice related to unaccompanied minor (UAM) treatment and also clinicians’ understanding of the law as outlined in Circular No. 4/2004 [2] which pertains to the scope of acceptable treatment for UAMs seeking primary care services. Respondents evaluated 10 scenarios to determine in which it would be reasonable for the UAM to receive care without prior parental notification or consent. A subsequent section detailed 6 different scenarios, querying in which it would be legally permissible for the clinician to retroactively notify parents about their UAM child’s condition and treatment.

Study results [1] indicated that “a vast majority of UAMs were in effect treated without parental consent.” While 3 in 4 respondents had been asked to treat UAMs in the prior year, more than half recalled grandparents accompanying minors, and only 1 in 4 recalled that UAMs usually presented for care alone. Reasons cited for parents not presenting with the child included: parents were too busy, family was comfortable with doctor due to long relationship, and parents perceived minor mature enough to present alone. Surprisingly, just over half of the respondents notify the parent after the visit despite the legal mandate to do so.

Israeli law mandates parental consent for treatment of minors 0–18 years of age. It does, however, permit minors to seek care without a parent/guardian in situations of “urgent need” and also “simple and ordinary treatment, which may be given where parents could not be located in a reasonable time frame [3].” It is unclear who determines what is urgent need, what is simple and ordinary, or what constitutes a reasonable time frame in which to locate a parent. Two additional categories are carved out, allowing Israeli minors to legally seek care without parental consent – HIV testing and pregnancy termination.

The study’s results suggest that clinicians are not well acquainted with this law despite the provision of a circular which aims to clarify its application to pediatric primary care [2]. No respondent correctly answered questions about all 10 scenarios regarding UAMs seeking treatment. Nor did anyone respond correctly to all 6 queries regarding when parents should be notified after particular UAM medical visits.

The Israeli approach appears to contrast subtly with practice in the United States regarding when physicians may and may not offer care to unaccompanied minors. First, each state defines its own parameters for UAM medical care [4]. The vast majority require parental consent for “simple and ordinary” medical care for anyone under age 18, which contrasts with the Israeli situation in which illness care, if deemed “simple and ordinary” or “urgent” (which is not further defined) may be offered and treated without first obtaining parental consent [2]. To consider one example-- the state of Michigan permits adolescents to seek care for sexually transmitted infections, family planning services, mental health, and substance use concerns without parental consent, and in an emergency situation, medical care may (and should) be provided even if parental consent cannot first be obtained [5]. However, it is essential to note limitations that align with or contrast with the Israeli clinical setting. Similar to the Israeli situation, American physicians may see patients to evaluate and discuss mental health and substance use therapy but in most states they cannot prescribe medication for mental health or substance use disorders without parental notification. Physicians in the U.S. cannot order laboratory or imaging tests or administer vaccines to adolescents without parental consent; the authors imply there is more latitude for “simple and ordinary” testing in Israeli primary care. In contrast, pregnancy termination can be sought by Israeli teens without parental consent but cannot be performed without parental notification (or court appearance) in most, but not all, American states. It is also likely that in the U.S. parents will be notified by their medical insurance company about details of their minor child’s medical visit, as an “EOB” (explanation of benefits) is sent automatically after most clinical encounters or when medical charges are posted.

In this clinical context, it is important to weigh the risks and benefits of treating unaccompanied minors. What is expedient and convenient in the moment must be weighed against the goals of providing informed care and maintaining the patient’s and family’s trust. Practices may wish to anticipate this need and discuss with families what constraints state or national law impose in this regard so that parents may appropriately accompany or offer verbal or written consent for their adolescent children to receive care. As Bravender states in his 2004 study surveying American physicians on their willingness to see UAMs, “the threshold question is who can give consent for the care…. If the parent is unaware…there must be a legal basis for the adolescent to consent [6].”

In the U.S., pediatric primary care’s goal in the medical home setting is to deliver evidence-based, patient- and family-centered care. This team-based approach comprises, at minimum, the patient, parent/guardian, and physician. Recognizing the importance of graduated autonomy for adolescents, the American Academy of Pediatrics [7] recommends allotting private time for the doctor and teen to discuss confidential or sensitive concerns. The Academy also encourages parents to inform adolescents of any significant family history and prepare questions ahead of time so that the patient can begin the process of taking ownership of their medical care. However, most pediatricians recognize that young teens and even many older teens are not fully capable of autonomous medical decision-making. Thus, pediatricians do appreciate the input of parents and generally invite them to participate in their adolescent’s clinical encounter. Indeed, we cannot order antibiotics for strep throat, update immunizations, or sign off on a detailed high school sports physical screening form without a parent’s input and consent. This contrasts with the ability of Israeli primary care practitioners to treat simple or urgent conditions without first seeking parental consent, though it does not obviate the need to notify parents after the consultation.

School-based clinics and many community and academic practices may proactively obtain parental consent annually, such as at school orientation, for basic services including urgent visits and immunizations. Bravender’s results [6] highlight the tension between the desire of the adolescent to maintain confidentiality, legal parameters defining which issues permit UAMs to seek confidential care, and other mechanisms by which clinics may seek consent such as via phone or written note. One exemplary tool to use in framing a discussion about the limits and constraints of adolescent health care can be found on the University of Michigan’s Adolescent Health Initiative website [8].

## Conclusions

The competing issues of expediency, expectations of the UAM and his or her family, legal constraints, and limits of confidential care are universal challenges to providing care to adolescent patients. It is apparent that Israeli law offers primary care clinicians some latitude to do what they perceive to be “urgent” and in the patient’s best interest. American physicians remain bound by more stringent state-level laws that clearly limit medical care delivery without parental consent. Anticipatory communication in all adolescent health care settings will improve overall health care delivery to teens and maintain, or even consolidate, the triad team of patient, parent, and physician. This study’s findings suggest that more can be done to increase knowledge of the laws that apply to adolescent health care in order to best meet the adolescent’s medical needs within the constraints of the legal environment in which they are seeking care. Additional cross cultural comparisons may help to identify best practices from which all clinicians caring for adolescents may benefit.

## Abbreviations

EOB: Explanation of benefits
UAM: Unaccompanied minor
